# Perceived life threat in children during the COVID-19 pandemic: associations with posttraumatic stress, anxiety, and depressive symptoms

**DOI:** 10.1186/s13034-024-00725-z

**Published:** 2024-03-18

**Authors:** BreAnne A. Danzi, Jessica T. Kelly, Ellen A. Knowles, Evan T. Burdette, Annette M. La Greca

**Affiliations:** 1 Department of Psychology, University of South Dakota, 414 East Clark Street, Vermillion, SD 57069 USA; 2 University of Miami, Coral Gables, FL USA

**Keywords:** Perceived life threat, COVID-19 pandemic, Children, Posttraumatic stress, Anxiety, Depression

## Abstract

Defining children’s “trauma exposure” in the context of the COVID-19 pandemic has been a source of debate. Children were exposed to threatening messaging about COVID-19 but might interpret this information differently than adults. Perceived life threat (PLT), the belief that one’s life is in danger, has been identified as a robust predictor of posttraumatic stress symptoms (PTSS), and may be a better predictor of PTSS than actual life threat (ALT). This study investigated parent reports of children’s self-PLT (belief that they might die from COVID-19) and family-PLT (belief that a family member might die from COVID-19). The aims were to compare PLT to ALT, evaluate their associations with children’s psychological functioning, and identify risk factors associated with PLT. We hypothesized an association between PLT and children’s psychological functioning in the context of the COVID-19 pandemic. Parents (N = 140) reported on their child’s (*M* age = 9.81 years, 47% female) pandemic experiences, psychological functioning, and both self-PLT and family-PLT. Results revealed self-PLT for 10% of the children and family-PLT for 43% of the children, yet only 6% experienced ALT (i.e., they or their parent tested positive for COVID-19). Children with reported self- or family-PLT had higher PTSS, depressive symptoms, anxiety symptoms, and functional impairment compared to children without these reported beliefs. PLT, but not ALT, was associated with psychological outcomes. Children with only PLT had greater PTSS and impairment than children with ALT. There were differences in parental functioning and pandemic-related information/media exposure between children with and without PLT. Children’s perceptions, rather than objective experiences, may be more central to their psychological functioning. This has implications for screening for pandemic-related symptomatology in children as traditional trauma exposure measures may not adequately identify distressed children.

## Introduction

As many as 60% of children experience a potentially traumatic event, which involves actual or perceived threat to the life or safety of themselves or a person close to them [11, 35]. The COVID-19 pandemic involved perceived and actual life threat to safety on a global scale [18]. COVID-19 posed actual life threat in situations where the virus led to severe illness and death in some individuals. In addition, individuals varied in terms of how much they personally perceived themselves (or family members) to be likely to die from COVID-19 (i.e., perceived life threat [18]). The presence of perceived life threat is a known risk factor for posttraumatic stress symptoms (PTSS) in both adults and children [28, 37]. Early identification of risk for PTSS can facilitate timely intervention and mitigate the effects of trauma on child development and well-being [28]. The purpose of this study was to understand how perceived life threat may have influenced children’s psychological functioning in the context of the COVID-19 pandemic.

### Perceived life threat (PLT)

PLT refers to the belief that one’s life is in danger [17]. PLT is predictive of worsened functioning in children following exposure to a potentially traumatic event and is associated with increased PTSS and internalizing problems [28, 32]. In fact, multiple meta-analyses on risk factors for PTSS in children and adolescents have identified PLT as a prominent risk factor [28, 37]. For example, a meta-analysis of 25 potential risk factors across 64 studies in children ages 6–18 found that PLT yielded larger effect sizes than many other well-documented variables, such as age, race, sex, SES, trauma severity, and psychological problems in the children or parent [37]. A more recent meta-analysis of 32 studies on risk factors for PTSS in children found similar results for PLT, and also identified sex as having a moderating impact, such that studies with a greater number of females identified larger effect sizes for PLT [28]. Moreover, children’s PLT also predicts more persistent PTSS following traumatic events [27] and is associated with increased rates of co-occurring symptoms of anxiety and depression [23, 32]. Although much of the literature on PLT has largely focused on associations with PTSS, a study of hurricane-exposed children also identified PLT as being related to increased internalizing symptoms in children [32]. However, little is known about whether PLT is relevant in the context of a global pandemic and how such perceptions are associated with children’s psychological well-being. Further, the literature to date has primarily focused on children’s *self-PLT*, the perceived threat to their own life. Given that children in the pandemic worried more about family members than themselves [10], this study expands the literature by also examining children’s *family-PLT*, their perceptions of threat to a family member’s life.

### Actual life threat (ALT)

Another dimension of life threat is the actual, or objective, threat to life. ALT can have both immediate (e.g., home destroyed) and lasting negative effects (e.g., needing to live in a shelter, separated from community supports) on quality of life and psychological well-being in children [25, 26]. In the context of the pandemic, contracting the COVID-19 virus led to hospitalization, chronic health problems, and death in some individuals [5].

As is the case with PLT, ALT has also been found to predict increased risk for PTSS in children [28]. However, PLT does not always coincide with the presence of ALT [17]. Although a degree of objective life threat is necessary for the diagnosis of posttraumatic stress disorder [1], Pittman and colleagues (2020) found that PLT may be a better predictor of post-disaster internalizing symptoms in children compared to ALT. Additionally, heightened attention to threatening stimuli was found to predict poorer functioning and increased risk for the development of psychopathology [29]. This increased attention to threat is one possible mechanism driving the predictive relationship between PLT and poor post-traumatic functioning in children [29]. However, there is limited understanding of the effects of PLT versus ALT on children following potentially traumatic events. Thus, further investigation into the potential influence of PLT on child functioning and how the impact of PLT compares to ALT is warranted and was examined in this study.

### Risk factors for PLT: parental functioning and information/media exposure

In addition to establishing whether PLT is associated with poor child functioning during the COVID-19 pandemic, it is important to investigate risk factors that identify which children may be most vulnerable to experiencing higher rates of threat perception. There are myriad developmental, biological, and environmental vulnerabilities that may contribute to increased perceptions of danger in children, thereby inflating children’s risk for PTSS following trauma [7]. This study focused on parental functioning and information/media exposure as potential risk factors for children’s PLT.

Regarding family factors, during the COVID-19 pandemic, family stressors have been associated with increased perceptions of threat [19]. Parent behavior in particular can have a strong influence on children’s PLT, as children have been reported to experience greater fear about the pandemic if their parents modeled, through verbal endorsement or behaviors, their own fear [34]. Furthermore, parental psychological functioning and PTSS may affect children’s perceived threat related to the pandemic [19]. Thus, parental functioning is likely to be an important risk factor for child PLT.

Information/media exposure, meaning information obtained through digital environments such as social media or television or through offline interactions such as conversation, is also likely to influence PLT in children. Radanović et al. [34] found that exposure to negative COVID-19-related information through the media directly impacted children’s fear of the pandemic. During the pandemic, the frequency of social media use and media consumption overall increased greatly among children and adolescents [14]. More time spent on social media was negatively associated with child psychological well-being and was positively associated with increased stress and fear about the spread of the COVID-19 virus and the likelihood of being infected [14]. While social media use is a primary source of information leading to greater fear in children, the role of other forms of media (e.g., television), and other forms of information sharing (e.g., conversation) is less clear and was evaluated in the current study.

### Current study

Given the pervasiveness of potentially distressing messaging during the COVID-19 pandemic, it is important to understand how children’s self- and family-PLT may be associated with their psychological functioning. This study evaluated children’s beliefs that they might die from COVID-19 (self-PLT) or that a family member might die from COVID-19 (family-PLT) and their associations with children’s psychological distress. Our specific aims and hypotheses were as follows:

*Aim 1* Compare PLT to ALT (i.e., whether there was actual exposure to the COVID-19 virus). We hypothesized moderate overlap between the constructs.

*Aim 2* Determine if PLT is associated with children’s psychological functioning (PTSS, depressive symptoms, anxiety symptoms) in the context of the COVID-19 pandemic. We hypothesized that children with PLT would have greater symptom severity and impairment than children without PLT.

*Aim 3* Evaluate whether PLT or ALT has a stronger association with child psychological functioning. We hypothesized that PLT would exhibit a greater contribution to symptom severity than ALT, when controlling for the potential influences of age, sex, and race/ethnicity.

*Aim 4* Identify potential risk factors associated with PLT. We hypothesized that demographic variables (age, sex, race/ethnicity), parent psychological functioning, and pandemic-related information/media exposure would be associated with children’s PLT.

## Methods

### Participants

Parents (N = 140) reported on themselves and one child (N = 140) under the age of 18 years (*M* age = 9.81, *SD* = 4.67). Participants were recruited from a predominantly rural Midwestern state and a predominantly urban Southern state in the US. There were not differences between sites for demographic or study variables, aside from higher child anxiety symptoms for the rural Midwestern state and a higher percentage of Hispanic participants for the urban Southern state, which is consistent with the respective demographics of those states. See Table 1 for sample demographic characteristics.

**Table 1:** Sample demographic characteristics

	Parents		Children	
Characteristic	n	%	n	%
Sex (% female)	133	95	65	46
Race/Ethnicity				
Non-Hispanic White	95	68	90	64
Hispanic White	26	19	28	20
Non-Hispanic Black	8	5	8	5
Hispanic Black	1	1	3	2
Asian (Hispanic and Non-Hispanic)	4	3	2	1
American Indian/Alaskan Native	2	1	2	1
Hispanic multiracial	3	2	2	1
Non-hispanic multiracial/other	1	1	5	4
Parental education				
High school degree or equivalent	13	9		
Some college	39	28		
Bachelor’s degree	39	28		
Graduate degree	49	35		
Gross annual income (pre-tax)				
Under $49 K	30	21		
$50 K–$74 K	33	24		
$75 K–$100 K	34	24		
Over $100 K	43	31		

### Procedure

Participants were recruited between September and December of 2020. Recruitment methods included social media advertisements or boosted posts targeting parents, and through email listservs to schools, churches, military networks, and community-based organizations. Surveys were completed online using Qualtrics software. Participants completed a 20–30-min online survey about themselves and one child under the age of 18. Participants did not receive compensation for survey completion. This study was approved by the relevant university Institutional Review Boards.

### Measures

#### Life threat

*Perceived life threat* The assessment of PLT was drawn from the Hurricane Related Traumatic Experiences-Revised scale, which is a widely used measure [25]. Child self-PLT was evaluated using the item, “Did your child think he/she might die? (Yes/No)”. Child family-PLT was evaluated using the item, “Did your child think that a family member might get sick and die? (Yes/No).” It is important to highlight that this study is assessing the parent’s view of their child’s self- and family-PLT. Parent-report of child self-PLT has been a focus of prior research (e.g., [16]); this study expands the construct to include parent reports of family-PLT.

*Actual life threat* ALT (Yes/No) was defined as either the child or parent testing positive for COVID-19. This definition was chosen because both having the virus and being in close proximity to a parent with the virus would be considered threats to the child’s health and safety in the context of a pandemic [19]. Thus, in separate items, parents were asked to indicate whether they or their child were tested for COVID-19 and the test result (positive, negative).

#### Child functioning

*Posttraumatic stress* Child PTSS was assessed utilizing the UCLA PTSD Reaction Index for DSM-5, parent report (UCLA-RI-5; [33]). The UCLA-RI-5 is a widely-used, reliable, and valid 31-item measure. Responses about child PTSS symptoms are reported on a 5-point Likert scale (0 = *None* to 4 = *Most*). Items were anchored to the COVID-19 pandemic. Internal consistency was *a* = 0.96.

*Depression* Children’s depressive symptoms were assessed using the 10-item MDD scale from the parent report version of the Revised Children’s Anxiety and Depression Scale (RCADS-25; [6]). Parents reported on the frequency of their child’s symptoms using a 4-point Likert scale (0 = *Never* to 3 = *Always*). Internal consistency was *a* = 0.90.

*Anxiety* Children’s generalized anxiety-related symptoms were evaluated using a 9-item subscale of the Screen for Child Anxiety-Related Disorders (SCARED; [2]). Parents reported on symptoms their child may have experienced using a 3-point Likert scale (0 = *Not True or Hardly Ever True* to 2 = *Very True or Often True*). Internal consistency was *a* = 0.90.

*Functional impairment* Child functional impairment was assessed using eight items (Yes/No) from the UCLA-RI-5. These were used to evaluate whether symptoms impacted the child’s home life, school, interpersonal relationships, and development [33]. Internal consistency was *a* = 0.88.

#### Parental functioning

*Posttraumatic stress* Parents’ PTSS was assessed using the PTSD Checklist for the DSM-5 [38], a widely-used 20-item measure. Parents rated symptoms on a 5-point Likert scale (0 = *Not at all* to 4 = *Extremely*,[38]). Scores of 31–33 suggest possible PTSD. Items were anchored to the COVID-19 pandemic. The internal consistency was *a* = 0.94.

*Depression* The Patient Health Questionnaire 9 (PHQ-9) is a widely-used, 9-item measure evaluating parents’ depressive symptoms [21]. Parents rated depressive symptoms on a 4-point Likert scale (0 = *Not at all* to 3 = *Nearly every day*). Internal consistency was *a* = 0.89.

*Anxiety* Parent anxiety symptoms were evaluated using the Generalized Anxiety Disorder-7 (GAD-7), a 7-item measure assessing anxiety symptoms on a 3-point scale [36], 0 = *Not at all* to 3 = *Nearly every day*). The GAD-7 includes the following clinical cutoff scores: 0–4 = Minimal, 5–9 = Mild, 10–14 = Moderate, 15–21 = Severe [36]. Internal consistency was *a* = 0.94.

*Perceived stress* Parents’ perceived stress was assessed using the Perceived Stress Scale (PSS; [9]), a 10-item measure that asks parents to rate their perceived trauma-related stress on a 5-point Likert scale (0 = *Never* to 4 = *Very often*). Internal consistency was *a* = 0.49.

#### Other measures

*Demographics* Parent and child demographic information included sex, age, race, Latin/Hispanic origin, education level, and socioeconomic status. The race/ethnicity variables were coded as 1 = person of color and 0 = non-Hispanic White in analyses.

*Child information/media exposure* Child exposure to pandemic-related information and media was assessed to identify the frequency of exposure via digital media (television, social media) and via conversation. Parents were asked “How much does your child watch media coverage of the coronavirus on television?” and rated their television exposure on a 5-point Likert scale: 0 = *Not at all (did not view news related to virus)* to 4 = *A whole lot (more than 5 h per day)*. Parents were also asked, “How much does your child engage in social media activity related to the coronavirus?” and “How much do you talk with your child in general about the coronavirus?” using a 5-point Likert scale: 0 = *Not at all (Once per day or less)* to 4 = *A whole lot (Almost continually*).

### Data analysis

Statistical analyses were performed using IBM SPSS Statistics Version 25. Data for this study came from a larger sample (n = 312) that included both parents and non-parents; 156 were parents and thus were eligible for study inclusion. Because 16 did not complete the survey, they were omitted due to large amounts of missing data, resulting in 140 participants. Item level missing data (< 5%) was handled using mean imputation. Of note, 19 participants did not complete the PTSS measure for their child and an additional 11 did not answer the information/media exposure items; thus, a subset of the sample was used for those analyses. Power analyses indicated sufficient power to detect effects for study hypotheses. Prior to analyses, variables were checked for normality and the frequencies, means, standard deviations, and correlations for relevant study variables were evaluated.

The prevalence of children with self-PLT, family-PLT, and ALT were identified. For Aim 1, within the subgroup of children that had either PLT and/or ALT, children with *only* PLT were compared to those with ALT (some of whom may have also had PLT) on psychological outcome variables using analysis of variance. For Aim 2, analysis of variance compared children with and without PLT (self and family) for psychological outcome variables (PTSS, depression, anxiety, and impairment). For Aim 3, linear regression models (with demographics, self-PLT, family-PLT, and ALT) were used to examine associations with each of the psychological outcome variables while controlling for the other variables in the model. For Aim 4, analysis of variance compared demographic characteristics, parental functioning, and pandemic-related information/media exposure between children with and without PLT (for self and family PLT). Because the purpose was to understand risk factors associated with PLT, children with dual PLT and ALT were not excluded from analyses.

## Results

### Preliminary results

In the sample, 10% of children were reported by parents to have self-PLT due to COVID-19. Most of these children (86%) had never tested positive for the virus. ALT (defined in this study as the parent or child contracting the COVID-19 virus) was present in 6% of the sample: positive COVID-19 tests were reported for 2% of the children and 4% of the parents at the time of data collection.

We also assessed children’s family-PLT (i.e., perception that a family member might die from COVID-19). Specifically, 43% of the children were reported to have family-PLT. Of those reported to have family-PLT, only 22% also had self-PLT. In contrast, nearly all of the children with self-PLT (93%) were also reported to have family-PLT.

Table 2 presents the means and standard deviations for study variables, and bivariate correlations for continuous variables. Means for child and parent symptoms mostly fell within the normal or mild range, although parent stress fell within the moderate range. Parent symptoms were highly intercorrelated and child symptoms were moderately to highly intercorrelated, but correlations between parent and child symptoms were low.

**Table 2:** Means, standard deviations, and correlations for key study variables

	*M* (*SD*)	2	3	4	5	6	7	8
1. Child PTSS	9.25 (14.88)	0.74*	0.56*	0.76*	0.47*	0.37*	0.34*	0.33*
2. Child depression	4.18 (4.99)	–	0.62*	0.64*	0.37*	0.38*	0.34*	0.39*
3. Child anxiety	6.51 (5.29)		–	0.41*	0.25*	0.35*	0.29*	0.29*
4. Child impairment	3.50 (4.39)			–	0.38*	0.28*	0.33*	0.31*
5. Parent PTSS	16.52 (13.86)				–	0.77*	0.73*	0.68*
6. Parent depression	7.97 (5.94)					–	0.76*	0.77*
7. Parent anxiety	9.76 (6.19)						–	0.72*
8. Parent stress	18.14 (7.43)							–

*p < 0.01

### Aim 1: how does children’s PLT compare to ALT?

Only 6% of the children had ALT. Interestingly, there was little overlap between ALT and reported PLT. Only 14% of children with self-PLT had ALT and only 8% of children with family-PLT had ALT. The majority (86–92%) of children with self- or family-PLT had not had any direct experience with the COVID-19 virus.

Psychological outcomes were compared between children with ALT versus those with only self-PLT. Children with only self-PLT had higher PTSS (*M* = 37.00, *SD* = 26.79) compared to those with ALT (*M* = 2.75, *SD* = 4.27), *F*(1,9) = 6.16, *p* = 0.035 and also had higher trauma-related functional impairment (*M* = 10.71, *SD* = 6.34) compared to children with ALT (*M* = 2.50, *SD* = 3.70), *F*(1,9) = 5.47, *p* = 0.044. No group differences emerged for anxiety or depression.

### Aim 2: is PLT associated with children’s psychological functioning?

Children with reported self-PLT were compared to those without self-PLT on the following: PTSS, depression, anxiety, and functional impairment (Table 3). Children with reported self-PLT had higher PTSS, depression, anxiety, and impairment than those with no self-PLT.

**Table 3:** Comparison of psychological functioning between children with and without PLT

	PLT *M* (*SD*)	No PLT *M* (*SD*)	Comparison	η^2^
Self				
PTSS	31.56 (25.74)	7.33 (12.35)	Welch’s *F*(1,8.30) = 7.83, *p* = 0.022	0.180
Depression	7.93 (5.84)	3.77 (4.74)	*F*(1,138) = 9.26 *p* = 0.003	0.063
Anxiety	9.79 (6.18)	6.15 (5.08)	*F*(1,138) = 6.18, *p* = 0.014	0.043
Impairment	10.33 (5.63)	2.95 (3.80)	*F*(1,119) = 9.15, *p* < 0.001	0.197
Family				
PTSS	15.71 (16.80)	4.74 (11.50)	Welch’s *F*(1,81.96) = 16.38, *p* < 0.001	0.140
Depression	5.40 (5.02)	3.28 (4.81)	*F*(1,138) = 6.43, *p* = 0.012	0.045
Anxiety	8.75 (5.02)	4.84 (4.87)	*F*(1,138) = 21.54, *p* < 0.001	0.135
Impairment	5.48 (5.06)	2.10 (3.21)	Welch’s *F*(1,76.49) = 17.40, *p* < 0.001	0.145

Similarly, children with family-PLT were compared to children without family-PLT (Table 3). Children with reported family-PLT had higher PTSS, depression, anxiety, and greater functional impairment, compared to those without this concern.

Given that most (93%) of the children with self-PLT also had family-PLT, we also examined how children with self-PLT or family- and self-PLT compared to children with only family-PLT. The self- and self/family-PLT group was higher than the only family-PLT group for PTSS and impairment, but there were no differences for depression and anxiety (all *p*s < 0.001).

### Aim 3: association between type of life threat and child psychological functioning

Linear regression evaluated the contributions of the various forms of life threat (self, family, actual) to child psychological outcomes, while controlling for the other types of life threat and important demographic variables (see Table 4). Older child age was associated with greater psychological distress across all the variables, although being female or a person of color was unrelated to outcomes. Self-PLT and family-PLT were both uniquely associated with child PTSS and impairment. Self-PLT also was associated with child depression, whereas family-PLT was associated with child anxiety. ALT was not associated with any of the psychological outcomes.

**Table 4:** Life threat associations with child psychological functioning

Variable	*b*	*SE b*	β
PTSS		*R*^2^ = 0.27***	
Child demographics			
Sex	1.36	2.42	0.05
Age	0.52	0.26	0.16*
Person of color	1.92	2.55	0.06
Self-PLT	17.58	4.72	0.32***
Family-PLT	7.77	2.59	0.26**
ALT	− 7.69	5.22	− 0.12
Depression		*R*^2^ = .22***	
Child demographics			
Sex	− 0.39	0.78	− 0.04
Age	0.40	0.08	0.37***
Person of color	0.89	0.81	0.09
Self-PLT	3.64	1.36	0.22**
Family-PLT	1.13	0.83	0.11
ALT	0.36	1.66	0.02
Anxiety		*R*^2^ = 0.22***	
Child demographics			
Sex	1.14	0.82	0.11
Age	0.28	0.09	0.25**
Person of color	0.32	0.86	0.03
Self-PLT	1.88	1.45	0.11
Family-PLT	3.32	0.88	0.31***
ALT	− 0.25	1.77	− 0.01
Impairment		*R*^2^ = 0.29***	
Child demographics			
Sex	0.12	0.71	0.01
Age	0.17	0.08	0.18*
Person of color	− 0.22	0.74	− 0.02
Self-PLT	5.95	1.44	0.36***
Family-PLT	2.32	0.76	0.26**
ALT	− 1.11	1.64	− 0.06

For “sex,” female = 1. For “person of color” variable, person of color = 1 and non-Hispanic White = 0. ***p < 0.001, **p < 0.01, *p < 0.05

### Aim 4: risk factors associated with perceived life threat

In terms of demographics, there were no differences in age, sex, or race/ethnicity between children with self-PLT compared to those without self-PLT. Similarly, there were no demographic differences between children with family-PLT compared to those without family-PLT.

In terms of parental risk factors, children’s self-PLT was not related to parental PTSS, anxiety, stress, or depression (See Table 5). However, children with family-PLT had parents with greater PTSS (*F*[1,138] = 4.28, *p* = 0.040), anxiety (*F*[1,138] = 6.06, *p* = 0.015), and stress (*F*[1,138] = 6.47, *p* = 0.012). A similar trend was observed for parental depressive symptoms (*F*[1,138] = 3.40, *p* = 0.067).

**Table 5:** Factors associated with self- versus family-perceived life threat

	Self-PLT			Family-PLT		
	Yes M (SD)	No M (SD)	η^2^	Yes M (SD)	No M (SD)	η^2^
Parent functioning						
Parental PTSS	19.57 (14.95)	16.18 (13.76)	0.005	19.28 (14.76)*	14.44 (12.85)*	0.030
Parental depression	8.71 (4.94)	7.88 (6.05)	0.002	9.03 (5.18)	7.18 (6.37)	0.024
Parental anxiety	11.21 (5.75)	9.60 (6.23)	0.006	11.22 (5.35)*	8.66 (6.56)*	0.042
Parental stress	17.59 (7.31)	18.20 (7.47)	0.001	19.95 (6.51)*	16.79 (7.82)*	0.045
Information/media exposure						
Television	0.56 (0.73)	0.41 (0.62)	0.004	0.58 (0.61)*	0.29 (0.61)*	0.054
Social media	0.44 (0.73)*	0.13 (0.39)*	0.040	0.23 (0.47)	0.10 (0.39)	0.023
Conversation	1.22 (0.97)*	0.59 (0.70)*	0.055	0.81 (0.76)*	0.52 (0.70)*	0.040

*p < 0.05

Exposure to pandemic-related information and media content was also related to children’s self- and family-PLT, although results differed depending on the mode of content delivery. Specifically, children with self-PLT had greater pandemic-related social media activity (*F*[1,108] = 4.55, *p* = 0.035), but this was not the case for children with family-PLT. In contrast, children with family-PLT spent more time watching COVID-19 television coverage (*F*[1,108] = 6.20, *p* = 0.014) than children who did not have family-PLT; however, there was no difference in COVID-19 television coverage for children with and without self-PLT. Finally, both children with self-PLT (*F*[1,108] = 6.30, *p* = 0.014) and family-PLT (*F*[1,108] = 4.52, *p* = 0.036) spent more time in conversation about COVID-19 compared to those without PLT.

## Discussion

Although PLT has been identified as a robust predictor of PTSS in children affected by disasters and other traumatic events, it was unclear whether PLT was relevant in the context of the COVID-19 pandemic. The pandemic differed from other well-studied traumatic events (e.g., disasters, mass shootings) in that direct exposure to the threat (i.e., the virus) could result in a range of individual responses from asymptomatic to fatal. This ambiguity in threat, coupled with ubiquitous media coverage and the impact of pandemic-related shutdowns, created a unique environment for children. Consequently, this study examined how children’s perceptions of life threat related to COVID-19 were associated with their psychological functioning. Studies of child psychological functioning during the COVID-19 pandemic are limited and have focused on anxiety or depression with scant attention to traumatic stress (e.g., [13]). Further, while past research has focused on self-PLT, we also investigated the prevalence of children’s reported family-PLT and its association with child psychological functioning.

### Prevalence of PLT and comparison to ALT

Our findings revealed that far fewer children reportedly perceived that they might die from COVID-19 (10%) than that a family member might die (43%), and nearly all children with self-PLT also reportedly had family-PLT (93%). The higher prevalence of family-PLT is consistent with research showing that children worried more about a family member or friend being infected with COVID-19 than themselves [10].

Interestingly, at the time of data collection, most children with either self- or family-PLT had not tested positive for COVID-19, nor had their parent. This suggests that children’s perceptions of pandemic threat may have been influenced by factors other than direct viral exposure [4, 34]. Prior research demonstrates that PLT can be present in children or adults without ALT; for example, PLT was reported by 22% of adults who had not been present at the site of a bombing [17]. It is possible that children’s biased attention to threat could be one important factor contributing to their perceptions of life-threat. Biased attention to threat has been implicated in the development and maintenance of child anxiety [4] and might also contribute to children’s perceptions of life-threat, a fruitful avenue for further investigation. In addition, other variables, such as information/media exposure or family functioning, may play a role in children’s PLT, as discussed below.

### Life threat and psychological functioning

Children with reported self- or family-PLT had greater PTSS, symptoms of depression and anxiety, and functional impairment than children with no PLT. Importantly, children’s ALT was not related to their psychological functioning.

Additionally, older children had worse psychological functioning than younger children, consistent with other literature (e.g., [22]). No differences in psychological functioning were observed based on sex or race/ethnicity.

The association between self-PLT and poorer child functioning during the pandemic is consistent with findings from other types of traumatic events, such as hurricanes and tornadoes [23, 25, 27, 32]. Our findings replicate and extend the broader trauma literature in important ways by confirming that self-PLT is associated with PTSS and other psychological outcomes, and also by demonstrating that family-PLT is significantly and uniquely associated with these psychological outcomes. This latter point is especially important given that more than four times as many children were reported to have family-PLT than self-PLT,

Also important was our novel finding that the smaller group of children with self-PLT reportedly had more severe PTSS and impairment than those with family-PLT alone. Thus, both self-PLT and family-PLT may be risk factors for PTSS and impairment, although children with self-PLT may be at higher risk for poor psychological outcomes.

Interestingly, when considering self- and family-PLT jointly in terms of their association with symptoms of depression and anxiety, self-PLT emerged as relevant for depressive symptoms and family-PLT as relevant for anxiety symptoms. The association between self-PLT and depressive symptoms may be due to elevated levels of PTSS observed in the self-PLT group. For example, a systematic review and meta-analysis of risk factors for depression in trauma-exposed children [8] revealed that PTSS was by far the largest risk factor for children’s post-trauma depression. Furthermore, studies of children following natural disasters have identified children with concurrent symptoms of PTSS and depression to be at greater risk for poor outcomes over time than those with just PTSS alone (e.g., [23]). Taken together with our findings, this suggests that children with self-PLT may represent a particularly vulnerable population in the context of a pandemic.

In contrast, the family-PLT group skewed more toward anxiety symptoms, which perhaps reflected these children’s worries about family health. Connor et al. [10] reported on a large sample of children in the UK during the first year of the pandemic, finding that children’s greatest worry was that family and friends would catch COVID-19 (51.3%); by comparison, only 27% of children worried about catching the virus themselves. It is also possible that anxious parents modeled fears about family health, leading to children’s general anxiety as well as worries about family health; systematic reviews have identified parental modeling of fear as an important factor contributing to children’s anxious cognitions (e.g., [15]). Further study of processes underlying the connections between PLT and children’s psychological functioning is needed.

### Risk factors associated with self- and family-PLT

In terms of family risk factors, we found that parental PTSS, anxiety, and stress were associated with children’s reported family-PLT. It is likely that children’s observations of their parents’ psychological distress influenced their perceptions that their family members were in danger. Alternatively, parents who were more psychologically distressed may have perceived their children to have more concerns about family safety.

Interestingly, conversations about the pandemic were related to both self-PLT and family-PLT. Parents who discussed the pandemic more frequently at home may have heightened children’s perceptions of COVID-19 threat. This fits with Nimphy et al. [30] finding that parents who were more fearful of COVID-19 provided more threat-related information to their children and, in turn, that parental threat information was related to children’s greater fear of COVID-19. Our findings in conjunction with those of Nimphy et al. [30] highlight the important role that parents’ psychological health and communication behaviors likely play in children’s perceptions of threat and also their psychological functioning.

In terms of media use, greater pandemic-related social media activity was associated with children’s reported self-PLT whereas greater exposure to television coverage of the pandemic was associated with family-PLT. Considering that the self-PLT group had the most severe psychological symptoms, this finding contributes to a growing empirical literature showing that news conveyed through social media has a greater impact on children’s psychological distress than conventional television news [31]. Notably, child social media use has also been associated with increased pandemic-related stress and fears about the likelihood of infection [14]. Parental monitoring of children’s social media use in the context of a pandemic or other potential traumatic stressor may be an important protective factor for children’s psychological functioning, as awareness of the detrimental risks of social media use has become more prominent (e.g., [3]). Further attention to the role of social media in heightening fearful self-perceptions in children is desirable.

### Caveats and future directions

To our knowledge, this is the first study to address the issue of children’s perceived life threat in the context of a pandemic and to link such perceptions to their psychological functioning. It also is the first to examine potential risk factors for children’s perceptions of life threat. Nevertheless, several limitations should be noted.

First, data were collected during the first year of the pandemic, when restrictions (i.e., social-distancing, school closures) were in place and before a vaccine was available. It is possible that children’s PLT, and the factors influencing PLT, might have lessened or changed as the pandemic became more cyclical and treatable. Future research evaluating children’s perceptions and psychological functioning over time would provide a more complete picture of how children’s risk and resilience evolve over the course of a pandemic.

Second, obtaining parents’ reports of child perceptions and functioning was an important first step in documenting children’s reactions and assessing children across a broad age range. However, given documented discrepancies in various informants’ reporting of child psychological functioning (e.g., [12, 20]), future research should expand on these findings by assessing children directly and using a multi-informant approach. A multi-informant approach will serve to more accurately assess children’s symptomatology through self-report while also having collateral parental information on the child’s functioning.

Third, it is possible that the lack of association between ALT and children’s functioning was due to the limited nature of the ALT measure, which focused on whether the parent or child had tested positive for COVID-19. This focus is consistent with literature indicating that threats to parents/caregivers are particularly impactful on children and the language in the developmentally-sensitive version of the DSM-5 criteria for PTSD (e.g., “Learning that the traumatic event(s) occurred to a parent or caregiving figure.” [1]). However, future research might expand the assessment of ALT to include items that reflect the child knowing someone who was hospitalized or who lost their life due to COVID-19, among other possible indicators.

Fourth, we were unable to obtain an adequate assessment of how race and ethnicity were related to children’s functioning, given the small sample size for some ethnic/racial groups. Our sample was comprised of children from predominantly non-Hispanic White and Hispanic White backgrounds. As such, the findings are most generalizable to children from those backgrounds; further replication of our findings with more diverse samples would be important and desirable.

Finally, data were collected online, which was an essential strategy during the pandemic as participants could not be recruited through traditional in-person methods (e.g., flyers could not be hung in businesses and non-essential personnel were not allowed inside facilities due to social distancing mandates). Online strategies for subject recruitment have increased markedly in recent years due to their cost efficiency and ease of use [20]. Nevertheless, future research may cast a wider net in terms of also accessing parents and children through in-person community settings.

### Clinical implications

Our findings have important clinical implications. In particular, mental health providers should assess PLT when screening children for pandemic-related distress, as it is easy to assess and could be an important marker for identifying children with significant psychological distress. Further, strategies to buffer against the development of PLT may be beneficial for promoting child mental health. During the pandemic, parents faced many challenges, including how to provide children with pertinent information to keep them safe (e.g., wearing a mask, social distancing) while also safeguarding their mental health. Conversations between parents and children are important, but frequent discussion of the pandemic, particularly when parents have a high degree of fear and focus on negative information, can be detrimental to child mental health [30, 34].

Parents, particularly those feeling a great deal of distress, may benefit from using available evidence-informed resources to help structure discussions about the pandemic (e.g., [24]). Parents might also monitor and limit children’s pandemic-related television exposure and social media activity, which can increase PLT and be detrimental to children’s psychological health [14, 24, 34].

## Data Availability

The data that support the findings of this study are available on request from the corresponding author (BD).
